# A Nosological Exploration of PTSD and Trauma in Disaster Mental Health and Implications for the COVID-19 Pandemic

**DOI:** 10.3390/bs11010007

**Published:** 2021-01-08

**Authors:** Carol S. North, Alina M. Surís, David E. Pollio

**Affiliations:** 1 The Altshuler Center for Education & Research, Metrocare Services, Dallas, TX 75247-4914, USA; 2 Department of Psychiatry, The University of Texas Southwestern Medical Center, Dallas, TX 73090-9070, USA; amsuris@hotmail.com; 3 VA North Texas Health Care System, Dallas, TX 75216, USA; 4 Metrocare Services, Dallas, TX 75247-4914, USA; davpoll62@yahoo.com

**Keywords:** COVID-19, pandemic, disaster, psychiatric disorders, posttraumatic stress disorder, nosology, psychiatric diagnosis criteria, trauma, exposure

## Abstract

The coronavirus disease of 2019 (COVID-19) pandemic rapidly spread around the world, resulting in massive medical morbidity and mortality and substantial mental health consequences. Post-traumatic stress disorder (PTSD) is an important psychiatric disorder associated with disasters, and many published scientific articles have reported post-traumatic stress syndromes in populations studied for COVID-19 mental health outcomes. American diagnostic criteria for PTSD have evolved across editions of the manual, and the current definition excludes naturally occurring medical illness (such as viral illness) as a qualifying trauma, ruling out this viral pandemic as the basis for a diagnosis of PTSD. This article provides an in-depth nosological consideration of the diagnosis of PTSD and critically examines three essential elements (trauma, exposure, and symptomatic response) of this diagnosis, specifically applying these concepts to the mental health outcomes of the COVID-19 pandemic. The current criteria for PTSD are unsatisfying for guiding the response to mental health consequences associated with this pandemic, and suggestions are made for addressing the conceptual diagnostic problems and designing research to resolve diagnostic uncertainties empirically. Options might be to revise the diagnostic criteria or consider categorization of COVID-19-related psychiatric syndromes as non-traumatic stressor-related syndromes or other psychiatric disorders.

## 1. The COVID-19 Pandemic

The coronavirus disease of 2019 (COVID-19) pandemic, involving the novel coronavirus pathogen Severe Acute Respiratory Syndrome Coronavirus 2 (SARS-CoV-2), emerged in the Wuhan province in China and spread rapidly around the world in the first few months of 2020. The seriousness of this illness quickly became apparent with the emergence of many cases with serious and catastrophic morbidity and mortality. The human toll climbed to tens of millions infected and more than a million deaths worldwide before the end of the year. General awareness of serious mental health consequences associated with COVID-19 followed, and within just a few months, the academic literature swelled with articles describing mental health outcomes, including 3504 articles from a PubMed search on 27 November 2020 using the terms “COVID-19” and “mental health”. Thus commenced the most deadly pandemic in more than a century, overwhelming efforts to respond effectively by public health authorities and clinical and academic professionals.

In disaster typology, pandemics are classified as natural disasters. Natural disasters have a well-known potential to generate pervasive mental health casualties. The “signature” psychiatric diagnosis of trauma is post-traumatic stress disorder (PTSD) [1,2]. PTSD has been extensively studied in association with all types of disasters, and it is the psychiatric disorder most often found in association with disasters [1,3]. PTSD is reported in as many as one-third of the most highly exposed survivors of the most severe disasters in studies using rigorous diagnostic assessment methods [4,5,6].

Numerous scientific articles have been published from studies of post-traumatic stress syndromes as mental health outcomes of COVID-19. The research samples in these studies include infected, exposed, unexposed, and mixed exposure groups, including patients hospitalized for COVID-19 illness, front-line healthcare workers caring for patients with COVID-19 illness, and the general population during the pandemic. A specific PubMed search of the terms “COVID-19” and “PTSD” on 27 November 2020 found 22 studies, which were combined with three additional studies located from citations in review articles emerging from the search. This collection of a total of 25 studies (with results published in 26 articles) includes only COVID-19 studies that collected original data from research samples and measured and reported findings on post-traumatic stress syndromes and symptoms. Table 1 summarizes the main methods and findings of these studies. It can be seen in Table 1 that all of these studies used brief self-report post-traumatic stress symptom screening tools, most commonly the PTSD Checklist (PCL) and the Impact of Event Scale (IES); none used full diagnostic PTSD assessment measures. This research originated from many different countries, most commonly China (*n* = 15) but also Spain (*n* = 2), Italy (*n* = 2), USA (*n* = 2), Greece (*n* = 1), France (*n* = 1), South Korea (*n* = 1), England (*n* = 1), Ireland (*n* = 1), Canada (*n* = 1), and India (*n* = 1). As summarized in Table 1, this collection of studies reported prevalence rates of “PTSD” or post-traumatic stress symptoms ranging from 12% to 96% in hospitalized COVID-19 patients (four studies), 4% to 73% in healthcare workers (11 studies), and 3% to 67% of general populations (10 studies)

## 2. Essential Nosological Concepts for Conceptualizing Trauma-Related Psychopathology

A firm understanding of the nosology of trauma-related psychopathology is important for designing and conducting research on post-traumatic stress-related outcomes of pandemics in broad populations that may be adversely affected in many ways, not only through personal threat of infection but also by secondary stressors accompanying pandemics. The purpose of this article is to clarify the nosology and conceptualization of PTSD for appropriate application of this diagnosis to disaster mental health outcomes, specifically in relation to the recent COVID-19 pandemic, which has unique issues for this diagnosis. Because PTSD is the “signature diagnosis” of trauma and of vital importance to disaster mental health outcomes, a clear understanding of the disorder and its application to disaster incidents is critical to understanding this literature [31]. This article will outline the new complexities that the current diagnostic criteria of the American Psychiatric Association (Diagnostic and Statistical Manual of Mental Disorders, 5th edition, DSM-5) [32] for PTSD have added to these considerations.

There are three major concepts that are essential to the general understanding of the definition of PTSD: (1) objective definitions of trauma and other stressors, (2) objective definitions of exposure to trauma, and (3) subjective emotional and psychological responses to trauma [2,31]. The first two of these three elements (trauma and exposure) constitute the two requirements of criterion A for PTSD. All three elements, including the symptoms (criteria B–E with additional symptom requirements in criteria F–H in DSM-5) anchored to the trauma exposure, are required for the diagnosis. To think clearly about trauma-related psychopathology, it is critical to understand each of these three entities and conceptualize them separately from one another, as confounding these concepts leads to logical problems that will be addressed below in detail.

### 2.1. Traumatic Stressors

PTSD is conceptualized and categorized as a trauma-related disorder. It is a conditional diagnosis specifically requiring the experience of trauma as defined in criterion A [2,31,32,33,34,35,36]. If the traumatic event is interpreted to be causal of the disorder, then the criteria for this diagnosis represent an exception to the general agnostic and atheoretical approach to diagnosis in the American Psychiatric Association’s diagnostic system that avoids the invocation of a potential etiology as part of the criteria [33,37,38]. Of note, however, the DSM-5 criteria removed causal language (“stressor producing” the disorder, and symptoms “resulting from” the traumatic event), and thus, causal assumptions might be avoided [33].

Trauma is narrowly defined in criterion A of the DSM-5 diagnosis of PTSD as a threat to life or limb: “actual or threatened death, serious injury, or sexual violence” (p. 271) [32]. Examples of trauma include blows causing lacerations, fractures, contusions, amputations, and rape [2,31,33]. According to DSM-5 criteria, qualifying types of trauma for consideration of PTSD include violence and accidents, such as physical assault, military combat, serious motor vehicle accidents, and disasters. Emotional reactions to the traumatic incident are not part of the criterion A definition of trauma and are discussed separately from the definition of trauma in separate criteria as the necessary symptom criteria for a diagnosis of PTSD. Thus, trauma is an objectively defined construct in the psychiatric nomenclature, not to be conflated with individual subjective reactions to an incident.

The DSM-5 definition of trauma is very different from the meaning of the word “trauma” used in common parlance. Standard dictionary definitions of the word trauma do not limit it to the experience of physical injury but also refer to psychological pain and disordered mental states resulting from severe mental or emotional stress or physical injury (https://www.merriam-webster.com/dictionary/trauma; https://www.thefreedictionary.com/trauma). This common usage definition of trauma is much broader than the psychiatric definition and could potentially include almost any experience that is very unpleasant, emotionally upsetting, or deeply distressing. Because the word “trauma” takes a far narrower definition in psychiatric nomenclature than in common language, careful reading of the DSM-5 criteria for PTSD is needed to comprehend that the psychiatric definition of trauma itself does not encompass psychological wounds that may arise from it.

The narrow DSM-5 definition of trauma also specifically does not include other non-traumatic stressors, and thus, trauma is a specific subset of a broader category of stressors. Experiences classified as stressors but not as trauma include various adversities and difficult or challenging incidents or situations not involving threat to life or limb, such as failure of an important examination, being served with divorce papers, or suffering a major financial loss. These are the types of stressors that might be associated with emotional or psychological pain or injury only, without physical wounds.

The DSM-5 definition of trauma is not a function of psychiatric symptom severity. The severity of the subjective response does not qualify a stressor as a traumatic event, which must be determined by objective criteria without regard for subjective reactions to it. Some non-traumatic stressors might evoke more severe symptoms than do some traumatic events; additionally, symptoms arising in the absence of any particular stressor might well be more intense and disabling than those following exposure to qualifying traumatic incidents.

### 2.2. Exposure

Conceptually distinct from the definition of trauma is the matter of exposure to it. The occurrence of trauma is a necessary condition for an individual to be exposed to a traumatic event, but it is not the same thing as exposure. When a traumatic event occurs, people must be exposed to it to experience its consequences. For example, people who are located in Houston or Los Angeles or London would not be directly exposed to a disaster that happens far away such as in New York City, as in the 11 September 2001 attacks that occurred there. The most obvious way to be exposed to a traumatic event is by being directly endangered, such as being in a motor vehicle accident, being shot or stabbed, or falling off a cliff. Such exposures may lead to both physical injuries and psychological sequelae. Other types of exposure such as witnessing trauma to others from a safe location or learning of a traumatic event experienced by a loved one, of course, do not result in physical injuries to oneself (only to others) but could, nevertheless, have emotional and psychological consequences [32]. It should also be noted that not everyone who experiences a disaster is necessarily exposed to trauma in that disaster. For example, a person’s city might be destroyed by massive flooding without that individual being personally exposed directly or indirectly to injury or endangerment or being a direct witness to it.

Through the evolution of versions of the criteria for PTSD from DSM-III through to DSM-IV-TR, the definitions of trauma and exposure to it have historically been jumbled together in the criteria without clear separation into the elements that define trauma and those that define exposure to it [33]. In DSM-5 criterion A, the words “exposure to” precede the trauma definition, contaminating the definition of trauma with exposure. This mixing of exposure and trauma in criterion A also separated the introduction of exposure from the subsequent description of the four exposure types (criteria A1–A4) by the definition of trauma placed between them. It would have been clearer if the criteria specifically addressed trauma first by itself and then separately discussed and enumerated the definitions of types of exposures to the trauma, rather than entangling the two concepts together in the introductory line of criterion A.

The distinction between trauma and exposure to it is critically important to the diagnosis of PTSD, which requires not only a traumatic event but also exposure to it. Thus, conceptualization of trauma and exposure independent of one another is needed to ensure that both are present for accurate diagnosis of PTSD [2,31]. For example, PTSD cannot occur with exposure to a non-traumatic stressor, nor can it occur in a person who is geographically distant from a mass casualty incident involving victims unknown to the person. Erroneous conflation of trauma and exposure to it can lead to enormous practical public health consequences. For example, unwarranted attribution of symptoms to PTSD in large trauma-unexposed populations can lead to overestimation of population prevalence of PTSD on the order of 10 times the magnitude and potentially millions of cases [2,39,40]. This can result in misdirection of resources for other needs that may be particularly strained in the post-disaster setting and to devaluation of the diagnosis of PTSD.

### 2.3. Subjective Emotional and Psychological Responses

Psychiatric symptoms and distress arising in the context of a traumatic event are distinct from the event and the exposure to it, but they are, by definition, connected. The symptoms must be anchored in a qualifying exposure to trauma, specifically beginning or worsening after the event to be considered post-traumatic stress symptoms. Such symptoms are referred to as incident symptoms. These are in contrast to all post-disaster prevalence of symptoms, many or most of which may be pre-existing and not representing disaster outcomes.

Psychiatric symptoms not anchored in a qualifying exposure to a traumatic event are not considered post-traumatic, such as symptoms developing after exposure to stressors not qualifying as trauma. Additionally, symptoms that were well entrenched before the traumatic event do not qualify. Thus, not all symptoms present after exposure to a traumatic event count as post-traumatic. Finally, psychiatric symptoms not following exposure to any particular event are simply symptoms.

## 3. COVID-19 and PTSD

The three nosological elements that are essential for understanding trauma-related psychopathology (objectively defined trauma, objectively defined exposure, and subjectively reported reactions) have unique considerations for COVID-19. Each of these three elements will next be considered separately in the context of COVID-19.

### 3.1. COVID-19 and Trauma

COVID-19 is a naturally occurring infectious disease. Despite the extensive human harm and death wreaked by the current pandemic, naturally occurring illness including COVID-19 does not constitute a qualifying trauma for the DSM-5 definition of PTSD, as stated by the text accompanying the criteria: “A life-threatening illness or debilitating medical condition is not necessarily considered a traumatic event” (p. 274) [32]. Thus, naturally occurring illnesses, including medically severe and even immediately life-threatening acute heart attacks and strokes, do not qualify as trauma for a diagnosis of PTSD given this constraint. Unfortunately, a complete delineation of the types of events that qualify as trauma was not provided in the actual criteria; the accompanying text description listed a few examples buried in the descriptions of exposure types, and none of the examples were naturally occurring illness (other than two noteworthy, stated exceptions: sudden and catastrophic hemorrhage of one’s child and anaphylactic shock). However, the diagnostic criteria for traumatic events qualifying for indirect exposure through close family members or close friends state that they are restricted to events that are “violent or accidental” (p. 271) [32].

Of historical interest, the accompanying text to the DSM-IV/-TR criteria directly conflicted with the DSM-5 exclusion of naturally occurring illness from categorization as trauma, specifically including “being diagnosed with a life-threatening illness” in the accompanying text only (pp. 424, 464) [41,42]. Thus, this about-face in the choice of categorization of naturally occurring medical illness as trauma between DSM-IV-TR and DSM-5 represents a dramatic change in the potential for pandemics to be considered as sources of PTSD.

The severity and potential lethality of COVID-19 do not qualify it as trauma by the DSM-5 definition because naturally occurring medical illness does not qualify even if severe and with potential for rapid fatality. It is thus categorized as a severe medical stressor, similar to acute heart attacks and strokes which are not considered traumatic events even if rapidly fatal. Not even the natural disaster classification of the COVID-19 pandemic can confer trauma status to SARS-CoV-2 infection or COVID-19 illness (this being a specific COVID-19 example of the earlier statement in the general discussion of exposure that not everyone who experiences a disaster is necessarily exposed to trauma in that disaster). The COVID-19 pandemic also generated important secondary stressors in the form of economic, social, and recreational losses that included closures of businesses and loss of jobs, curtailed opportunities to socialize with friends and loved ones, and discontinuation of many of life’s usual pleasurable or meaningful activities [43], further augmenting the severity and the breadth of the pandemic on affected populations.

Because viral infection in the COVID-19 pandemic is not technically classifiable as a trauma by current criteria, it is not plausible to contemplate it as a substrate for PTSD, even though the COVID-19 pandemic is considered to be a disaster. The reported findings of COVID-19-related post-traumatic stress (PTSD, “probable” PTSD, post-traumatic stress symptoms and syndromes) in the published COVID-19 literature are not consistent with the DSM-5 diagnostic convention. Because the COVID-19 studies measuring post-traumatic stress have nosological problems in the conceptualization of trauma, it follows that the post-traumatic stress syndromes and symptom outcomes reported in these articles do not technically conform to DSM-5 criteria for PTSD. Five of the 25 studies summarized in the current article acknowledged methodological limitations in the assessment of post-traumatic stress symptoms using self-report symptom measures that do not provide diagnostic assessment of PTSD [13,18,22,24,44]. This is an important methodological problem that has been well established in the research literature [45]. One article acknowledged inability to distinguish new (incident) stress symptoms from pre-existing stress symptoms in its research methods [20], and one study acknowledged the use of a symptom measure not referenced to the COVID-19 experience [25], further departing from the identification of mental health outcomes related to the pandemic. None of these studies considered that the DSM-5 criteria for PTSD do not include naturally occurring medical illnesses such as a viral infection as representing a qualifying traumatic event, and reference to the pandemic was generally broader than the specific aspect of viral exposure. One article curiously concluded that its results “suggest that the COVID-19 pandemic could be considered as a traumatic event” (p. 1) based on its findings of subjective mental health effects, inconsistent with the stated DSM-5 exclusion of naturally occurring medical illness as a qualifying trauma [25].

### 3.2. COVID-19 and Exposure to the Virus

Because SARS-CoV-2 is classifiable as a stressor but not trauma, exposure to this non-traumatic stressor as well as to the secondary stressors arising from the pandemic is pertinent to the categorization of the ensuing emotional and psychological consequences of COVID-19. Estimates of COVID-19 post-traumatic stress reported in the published literature, thus, do not reflect trauma-related symptoms or syndromes but rather stressor-related symptoms or syndromes. COVID-19 studies have the potential to reintroduce the same methodological errors in estimating adverse mental health consequences described earlier in studies of disasters in very large unexposed populations, first by measuring post-traumatic symptoms, which is not technically justified given the non-traumatic stressor status of viral infection, and second by sampling viral-unexposed populations.

In the published COVID-19 research literature, the different exposure characteristics of the three populations sampled (patients with COVID-19 illness, healthcare workers, and general populations) and potential variability in individual exposure within these populations makes it difficult to compare findings across studies and to understand what the widely differing rates reported actually reflect. Samples of patients hospitalized for COVID-19 illness were all clearly exposed to COVID-19. In the general population studies, very few individuals likely had SARS-CoV-2 exposure or infection, an assumption based on the small fractions of general populations worldwide known to have been exposed or infected (https://www.nytimes.com/interactive/2020/us/coronavirus-us-cases.html). Most of the COVID-19 studies of general populations reported far higher proportions with COVID-19-related “post-traumatic stress” in their samples than the proportions with known SARS-CoV-2 exposures, indicating conceptual problems in the research design and reporting. In the COVID-19 studies of healthcare workers, the exposure status of the workers was likely mixed and more complex. For healthcare workers, when personal protective equipment (PPE) is used and it functions properly, the worker wearing it is not bodily exposed to the virus, which stops, as intended, outside the PPE. Only when the PPE is not used, is not used properly, or fails is the worker physically exposed to the virus. Because the virus is imperceptible, healthcare workers may not be able to discern personal exposure to the virus unless they test positive for it or develop COVID-19 illness symptoms. The proportions of healthcare workers who were personally exposed to SARS-CoV-2 in caring for patients with COVID-19 illness in these studies are unknown and may have been diminutive, creating further validity problems.

Among virus-exposed individuals, the most minimal end of the exposure spectrum may include physical contact with the virus not leading to infection, but this does not negate the exposure. (There may be some situations in which it is not certain as to whether an exposure has occurred, such as having contact with someone exposed to the virus who may or may not have viral infection and thus may or may not be a source of personal exposure to the virus. Until the contact is known to carry the virus, an exposure cannot be determined and thus the incident technically cannot be classified as an actual exposure). More severe exposure may be experienced in higher levels respectively among individuals testing positive for infection with the virus but not becoming symptomatic. Among those individuals who do develop COVID-19 illness, the medical illness itself constitutes yet another dimension of exposure severity. Among those who become medically ill, in turn, different levels of medical effects ensue, including illness requiring medical care and more severe illness requiring intensive care and even ventilator assistance. These considerations suggest that not only does exposure to the virus have dose-related direct effects on both medical complications of the viral illness and mental health, but the severity of the medical illness may serve to further drive more severe emotional and psychological consequences. Besides direct personal exposure, other types of exposure to the virus may prove stressful, including witnessing the course of severe COVID-19 illness in previously healthy patients, learning about severe COVID-19 illness in a close family member or a close friend, or even having personally exposed or infected a close family member or close friend.

Potential for exposure is a major source of worry, fear, and anxiety that has been extensively articulated by medical professionals and members of general populations during the COVID-19 pandemic. The DSM-5 criteria for PTSD do not include risk of exposure to trauma as a qualifying exposure type [32]. Risk of exposure with the fears that surround it is conceptually different from actual exposure to the virus because it represents only a probability or a possibility rather than an exposure that is known or knowable to the individual. Fear of exposure also introduces a subjective dimension not consistent with objective definitions of exposure.

Regardless, fears of exposure create emotional burdens for both healthcare workers and general populations. People affected by concerns about risk for exposure constitute the largest segment of the population and could include almost everyone not exposed to the virus but who, nevertheless, find the pandemic to be stressful, anxiety-provoking, and fear-inducing. (These would ostensibly not include people who willingly attend super-spreader events or choose not to use precautions such as physical distancing or masks). These widespread concerns about risk of exposure are of potentially limitless dimensions and might even be at least as distressing as the specific and contained awareness of an actual and identifiable virus exposure, as in a popular saying, “Fear of the unknown is the greatest fear of all” (attributed to Yvon Chouinard, outdoor adventurer and founder of the clothing and gear company Patagonia). Particularly in the initial phases of the pandemic when less was understood about the virus and its transmission, realistic assessment of the level of risk from daily activities for potential exposure to the virus, infection, and medical illness and complications was particularly difficult. Initial research on the psychological effects of the COVID-19 pandemic conflated actual exposures with concerns about risk for exposure in various samples, consequently mixing mental health consequences of actual exposures to the virus with concerns about potential exposure.

### 3.3. COVID-19 and Emotional/Psychological Outcomes

Examination of published COVID-19 research can illustrate the importance of addressing the objective elements of trauma and exposure to it separately from, and in relation to, the subjective emotional/psychological responses. Because COVID-19 illness does not qualify as trauma according to DSM-5 criteria, mental health problems related to SARS-CoV-2 exposure cannot be considered to represent trauma-related syndromes, and PTSD—a post-traumatic syndrome—cannot be diagnosed with these criteria in these studies. Instead, COVID-19-related symptoms can more appropriately be characterized as representing stressor-related symptoms or syndromes.

Because psychological symptoms reported by people not exposed to SARS-CoV-2 cannot be attributed to trauma exposure according to the DSM-5 criterion A for PTSD, the reported post-traumatic stress syndromes in the published COVID-19 literature are technically not valid by these criteria. Because people without exposure to the virus cannot be considered to meet the DSM-5 criterion A for PTSD related to the pandemic, measurement of post-traumatic symptoms in trauma-unexposed individuals departs from the DSM-5 definition of post-traumatic stress syndromes by not satisfying both of the two required elements of criterion A: trauma and exposure to it. The self-report symptom tools used to collect data in the published studies either did not anchor the symptoms to COVID-19 exposure or inappropriately ascribed symptoms related to SARS-CoV-2 exposure as trauma-related, not fulfilling the requirement in DSM-5 criteria that the symptoms must be anchored in exposure to a qualifying traumatic event to be considered post-traumatic stress symptoms. Non-diagnostic self-report symptom measures are well known to have these inherent problems and contribute to overestimation of diagnostic prevalence. Therefore, these instruments cannot be used for diagnosis or estimation of population prevalence of post-traumatic stress arising from the COVID-19 pandemic [1,2,39,40,45,46].

Even though the mental health outcomes of COVID-19 in these studies did not have adequate methodological designs to identify post-traumatic stress as envisioned, importantly, they seem to have identified many symptomatic individuals. This growing body of research collectively points to substantive psychological and emotional reactions to the COVID-19 pandemic that may be not only distressing but also possibly including pathological outcomes. Inability to classify the mental health consequences as trauma-related psychopathology does not automatically render the mental health outcomes trivial or unimportant. Many more people experience distress than disaster-related psychopathology, representing more opportunities for distress intervention than for formal treatment of trauma-related psychopathology.

## 4. Toward Resolution of Psychiatric Categorization Dilemmas in COVID-19-Related Syndromes

Because naturally occurring medical illness such as viral infection does not represent a source of PTSD according to DSM-5 criteria, a pertinent question emerges. Among those directly exposed to or infected with SARS-CoV-2, if the reported psychiatric symptoms are not manifestations of PTSD, what do they represent? There are several possibilities for addressing this dilemma in the diagnostic nomenclature.

First, if the criterion A definition of trauma could be rewritten to allow naturally occurring serious medical illness (including viral infection) as a qualifying trauma, then COVID-19 illness could lead to a diagnosis of PTSD. However, inclusion of a broad collection of medical illnesses in the definition of trauma could widen its definition so far as to make it meaningless. It might create difficulties with decisions of which illnesses to include, such as COVID-19, influenza, (certainly HIV, especially in years before effective treatments were developed) cancer, or even diabetes mellitus, all of which significantly increase an individual’s risk of fatality. Additionally, no one survives life without eventual fatality, and thus, even birth itself could be conceptually construed as meeting criteria for trauma by this logic. None of these medical conditions conform to the stated rule that they must not only be potentially life-threatening but also sudden and catastrophic. Including these medical illnesses in the PTSD definition of trauma might introduce syndromes that are very different from the PTSD defined by the DSM-5 criteria.

Alternatively, it is possible that the psychiatric symptom response to SARS-CoV-2 exposure or COVID-19 illness represents a yet-undefined stress-related syndrome similar to PTSD based on exposure to a stressor not considered a trauma. This possibility could lead to a new diagnostic entity for psychological syndromes associated with acute life-threatening medical illnesses that is not currently available in the DSM-5 criteria. This could potentially avoid the proverbial dubious attempts to fit a square peg into a round hole by inserting a diagnostic entity into the PTSD definition that is fundamentally not the same disorder. Potential solutions involving new diagnostic entities, however, are not without potential problems. First, the longitudinal trend in successive versions of the diagnostic manuals has been to greatly expand the numbers of established psychiatric diagnoses [38], yielding diminishing utility of a system that has far more diagnoses than most clinicians would ever use or necessarily even be generally aware of. Second, such new diagnostic entities would certainly face the same issues with determining the boundaries of inclusivity of medical illnesses that fit the criteria of the chosen diagnostic category. Third, decisions would be needed on whether to create separate psychiatric diagnoses for different medical illnesses or whether diagnostic subcategories of one main medical illness-related stress-induced disorder might be more appropriate.

Another possibility might be that the psychological syndrome related to SARS-CoV-2 exposure or infection or COVID-19 illness might actually be part of another well-established psychiatric illness such as anxiety or depressive disorders, which have been well documented [47]. Such disorders may include both pre-existing (either continuous or recurrent) disorders and disorders arising for the first time (incident disorders). It is well known that depressive and anxiety disorders may occur in the context of difficult life circumstances and stressors, which could include COVID-19.

Yet another possibility is that the psychological syndrome related to SARS-CoV-2 exposure or infection or COVID-19 illness could represent emotional distress not rising to the level of diagnosable psychopathology. Such distress is well documented by research to be nearly universally reported after exposures to severe trauma such as disasters [48,49,50,51]. Similar to more general trauma studies that have demonstrated a dose-related mental health response in relation to the severity of the trauma exposure [48,49,50,51], it would be expected that people with more severe SARS-CoV-2 exposure (e.g., resulting in severe and life-threatening COVID-19 illness) on average would generally report more severe and prevalent mental health outcomes.

Much of the focus of the discussion of COVID-19-related concerns in this article has been on post-traumatic stress-related outcomes of known or confirmed exposure to the virus. If the DSM-5 criteria for PTSD were rewritten to include risk of exposure as a qualifying trauma exposure, then simply having potential for exposure could lead to a diagnosis of PTSD. This solution would have the same issue with naturally occurring medical illness not meeting the criteria for categorization as trauma in addition to the lack of actual exposure. Expansion of PTSD criteria to include fear of exposure as a qualifying trauma exposure could be expected to render the criteria meaninglessly broad. For example, just as concerns about risk for trauma exposure in general could be extended beyond reason to include simply driving on a highway, concerns about risk for COVID-19 could be extended beyond reason to include simply taking a breath of fresh outside air from an open window. Nevertheless, the substantial proportion of general populations and healthcare workers reporting psychiatric symptoms related to concerns about potential viral exposure in published COVID-19 research studies supports the concept of concern about risk for viral exposure as an important stressor with potential for mental health consequences. Research might find the mental health outcomes related to fear of exposure to also represent stressor-related disorders, other psychiatric illness such as depressive and anxiety disorders, and non-pathological distress, with all the same potential issues for diagnostic categorization discussed above for categorization of viral exposure.

## 5. Discussion

This article’s review of disaster-related PTSD has addressed nosological issues of this diagnosis specific to the current COVID-19 pandemic. The issues rising from the DSM-5 diagnostic criteria for PTSD are unsatisfying for guiding the response to this disaster because the constraint of its definition of trauma, which excludes natural illness such as viral infection, has not been fully investigated. The international criteria do not include this constraint, and supporting evidence for this new change in the American diagnostic criteria is needed. To determine whether exposure to the SARS-CoV-2 virus represents a qualifying traumatic event or a non-traumatic stressor will require empirical data. Research must be designed to compare SARS-CoV-2 exposure to exposure to qualifying traumatic events according to DSM-5 criteria to determine whether the symptomatic response to both types of events is similar or distinct.

A time-honored approach to differentiation of disorders from one another has been to invoke the five-phase validation procedure applied to psychiatric diagnoses by Robins and Guze [52]. Using this procedure, if the syndrome following exposure to SARS-CoV-2 displays a distinctive symptom complex and clinical characteristics, continues to manifest consistently over time with characteristic treatment outcomes, runs in families by itself, can be demonstrated by exclusion criteria to be identifiably different from other established illnesses, and especially if distinctive biomarkers can be found, then it suggests the syndrome following SARS-CoV-2 exposure may be distinct from syndromes following exposure to traditionally qualifying traumatic events. Conversely, if the COVID-19-related psychological syndrome appears equivalent to other trauma-related syndromes across these five procedural phases, this might be considered evidence that it represents a trauma-related syndrome and could lead to PTSD. Alternatively, this direction of research investigation could potentially help determine whether the COVID-19-related syndrome fits with known non-trauma stressor-related syndromes. Additionally, fears related to potential for exposure are a sufficient source of stress such that research is needed to further characterize the mental health effects of these concerns and differentiate them from mental health outcomes of the stress of actual viral exposures and of secondary stressors of the pandemic.

Just as the case has been convincingly made for the need for diagnosis in medical and psychiatric practice, it also applies to the categorization of psychiatric syndromes related to the COVID-19 pandemic. Accurate diagnosis is central to all of medical practice; it guides the clinician to select the most appropriate and effective treatment, helps determine a likely prognosis, allows communication with professional colleagues, facilitates education of clinicians and researchers, and organizes scientific research [53]. All of these activities have central relevance to mounting and executing the most effective responses to the COVID-19 pandemic. The specific relevance for the psychiatric aspects of COVID-19 regarding determination of whether exposure to the virus represents trauma and thus constitutes a source of PTSD in the traditional sense as currently defined by DSM-5 or a non-trauma stressor and stress-related syndrome is particularly salient for psychiatric treatment. For example, there are efficacious treatments for PTSD [54,55], but if the disorder is not the same as PTSD, the safety and efficacy of PTSD treatments may not be known. These concerns are relevant not just for the millions of people affected by the current COVID-19 pandemic but also for the broad populations that will experience threats of infection and other stressors arising with the upcoming epidemics and pandemics that the future will inevitably bring; thus, research addressing these issues is greatly needed for the current and future welfare of the world.

## Figures and Tables

**Table 1:** Studies of post-traumatic syndromes and symptoms associated with the COVID-19 pandemic (all published in 2020).

First Author	Sample/Recruitment/Participation	Data Collection Method	Assessment Tool	Results
**Patients hospitalized with COVID-19**				
Cai [7]	126 patients hospitalized with COVID-19 and cured (China)—participation rate unknown	Online survey	PTSD-SS	Clinically significant PTSD symptom score 31%
Qi [8]	41 patients hospitalized with COVID-19 (China)—39% participation	Self-administered questionnaire	PCL-C	“PTSD symptoms” 12%
Chang [9]	64 patients hospitalized with COVID-19 (South Korea)—60% participation	Telephone interview	PCL	“PTSD” 20%
Bo [10]	714 patients discharged from hospitalization for COVID-19 (China)—participation rate unknown	Online questionnaire	PCL-C	“Significant posttraumatic stress symptoms” 96%
**Healthcare workers**				
Chew [11]	906 mixed medical disciplines from 5 hospitals (Singapore; India); 91% participation	Self-administered questionnaire	IES-R25	“PTSD” 7%
Blekas [12]	270 mixed medical disciplines (Greece)—participation rate unknown	Online survey via social media	PTSD-8 questionnaire	“Probable PTSD” 17% (22% in women, 5% in men)
Si [13]	863 hospital clinical and administrative staff (China)—participation rate unknown	Online survey	IES-6	“PTSD” 40%
Luceno-Moreno [14]	1422 mixed medical disciplines providing COVID-19 care (Spain)—“non-probabilistic sampling”; participation rate unknown	Online survey	IES-R	“PTSD symptoms” 57%
Di Tella [15]	145 physicians and nurses (Italy)—convenience sample	Online survey	PCL-5	“PTSD symptoms” 26%
Wang [16]	202 nurses providing COVID-19 care (China)—random sampling; participation rate unknown	Self-report questionnaire	PCL-C	“PTSD incidence” 17%
Song [17]	14,825 ED medical staff (China)—snowball and convenience sampling	Electronic questionnaire	PCL-5	“PTSD” 9%
Yin [18]	377 mixed medical disciplines from contacts of investigators (China) —participation rate unknown	Online survey via email and social media	PCL-5	“Posttraumatic stress symptoms” 4%
Liu S [19]	1563 medical staff (China)—participation rate unknown	Hospital survey	IES-R	IES-R trauma-related distress 73%
Lai [20]	1257 physicians and nurses (China)—“cluster sampling”; 69% participation	Hospital survey	IES-R	IES-R trauma-related distress 72%
Caillet [21]	208 ICU workers of mixed healthcare disciplines (France)—95% participation	Hospital survey	IES-R	“PTSD” 27%
**General population**				
Chi [22]	2038 students of >180 universities (China)—participation rate unknown	Online survey via social media and websites	Abbreviated PCL-C	“Clinically relevant PTSD symptoms” 31%
Li [23]	1109 community participants (China)—participation rate unknown	Online survey	IES-R	“High PTSD level” 67%
Karatzias [24]	1041 members of general population (UK and Ireland)—“stratified quota sampling”; participation rate unknown	Email survey	ITQ	“COVID-19-related PTSD” 18%
Forte [25]	2286 general population (Italy)—participation rate unknown	Web-based survey via social media	PCL-5-based COVID-19-PTSD questionnaire	“PTSD symptomatology” 29%
Liu C [26]	898 members of general population, adults aged 18–30 years (USA)—volunteer sample	Online survey via websites and social media	PCL-C	“PTSD symptoms” 32%
González-Sanguino [27]	3480 members of general population (Spain)—snowball sampling	Online survey via social media	PCL-C-2 (reduced version)	Traumatic stress symptoms 16%
Liang [28]	584 members of general population, aged 14–35 years (China)—snowball sampling	Online questionnaire via social media	PCL-C	“PTSD symptoms” 14%
Liu N [26]	285 adult city residents (China)—participation rate unknown	Internet survey	PCL-5	“Posttraumatic stress symptoms” 7%
Taylor [29]; Taylor [29]	6854 “population representative sample” (USA, Canada)—recruited by web-sampling; participation rate unknown	Internet self-report survey	36-item COVID Stress Scales	“COVID traumatic stress” 16%
Tang [30]	2485 home-quarantined students of 6 universities (China)—convenience sample	Online survey	PCL-C	“Probable PTSD” 3%

PTSD = post-traumatic stress disorder; COVID = coronavirus disease; HCW = healthcare worker; IES = Impact of Event Scale; PCL = PTSD Checklist; ITQ = International Trauma Questionnaire; ED = emergency department; UK = United Kingdom; USA = United States of America; -SS = self-rating scale; -C = civilian; -R = revised.

## Data Availability

Not applicable.
